# Hepatitis B and C viral coinfections and their association with HIV viral load suppression among HIV-1 infected patients on ART at Mekelle hospital, northern Ethiopia

**DOI:** 10.1186/s12981-022-00479-8

**Published:** 2022-12-01

**Authors:** Gebrecherkos Teame, Araya Gebreyesus, Ephrem Tsegay, Mulu Gebretsadik, Kelemework Adane

**Affiliations:** 1 Tigray Health Research Institute, Mekelle, Ethiopia; 2 Department of Microbiology and Immunology, Division for Biomedical Sciences, College of Health Science, Mekelle University, Mekelle, Ethiopia; 3 Department of Clinical Laboratory, Ayder Comprehensive Specialized Hospital, Mekelle, Ethiopia; 4 Department of Microbiology, Immunology, and Parasitology, School of Medicine, College of Health Sciences, Addis Ababa University, Addis Ababa, Ethiopia

**Keywords:** Antiretroviral therapy, HBV, HCV, HIV, Viral load suppression

## Abstract

**Background:**

Although Ethiopia is endemic to viral hepatitis and HIV, data that could guide population-specific interventions are limited. In this study, we determined the seroprevalence of hepatitis B virus (HBV) and hepatitis C virus (HCV) and assessed their associations with HIV-1 viral load suppression among HIV-1 infected patients on antiretroviral therapy (ART) at Mekelle hospital in northern Ethiopia.

**Methods:**

Between February and April 2020, blood samples were collected from 439 participants. Samples were screened for HBsAg and anti-HCV on the immunochromatographic test and confirmed using the Enzyme-Linked Immuno-sorbent assay (Beijing Wantai Co. China). HIV-1 viral load was quantified using reverse transcription-polymerase chain reaction (RT-PCR) on the Abbott platform. Binary and multivariable logistic regression was performed to identify potential predictors.

**Results:**

Overall, 10% (44/439) and 3.6% (16/439) of the participants were coinfected with HBV and HCV, respectively. In a multivariate analysis, being illiterate (AOR = 6.57; 95% CI 1.04–41.6), and having a history of sexually transmitted infections (AOR = 4.44; 95% CI 1.31–15.0) and multiple sexual partners (AOR = 29.9; 95% CI 7.82–114.8) were associated with HBV infection. On the other hand, participants with a history of chronic non-communicable diseases (AOR = 10.6, 95% CI 1.61–70.1), and those reporting a history of sexually transmitted infections (AOR = 5.21, 95% CI 1.39–19.5) were more likely to be infected with HCV. In further analysis, HCV infection status was significantly associated with decreased viral load suppression rate (AOR = 7.14; 95% CI 2.18–23.3) whereas no significant association was observed with the HBV infection.

**Conclusions:**

The HBV coinfection rate in our study is high and, as per WHO's standard, corresponds to a hyperendemic level. The HCV coinfection rate is also substantially high and urges attention given its influence on the viral load suppression of HIV patients on ART at our study site. Our findings suggest the need to adopt universal screening and vaccination of people with HIV against HBV and screening for HCV at our study site and in Ethiopia at large, which contributes to Ethiopia's progress towards the 2030 global target of reducing the HBV infection.

## Background

Viral hepatitis is a group of infectious diseases that affect hundreds of millions of people worldwide. In particular, Hepatitis B virus (HBV) and Hepatitis C virus (HCV) are the predominant causes of chronic viral infections and become the second top killer of infectious diseases following tuberculosis. World Health Organization (WHO) reports that HBV and HCV cause 1.1 million deaths and 3.0 million new infections per year [1]. However, only 10% and 21% of people who have chronic infection with HBV and HCV are diagnosed, respectively [1]. Because of the shared modes of transmission, coinfections of HBV and/or HCV are common with Human Immunodeficiency Virus (HIV) infection.

While the use of antiretroviral therapy (ART) has significantly improved the life expectancy of HIV patients, hepatitis viral coinfections have become increasingly important [2, 3]. According to WHO, nearly 37.7 million people were living with HIV worldwide in 2020; the majority residing in low-and middle-income countries (LMICs) [1]. About 2.7 million and 2.3 million had HBV and HCV coinfections with the African and the southeast Asian regions taking the maximum share [4]. In sub-Saharan Africa, a systematic review and meta-analysis showed that 15% and 7% of the HIV cases were coinfected with HBV and HCV, respectively [5]. Untreated hepatitis coinfection promotes more rapid progression of hepatitis B– and/or C–related liver disease, hepatocellular cancer, and untimely death, undermining the gains of effective HIV treatment. Studies from some endemic countries have shown a slow rate of immunologic recovery and poor virologic response after initiation of ART among HBV and HCV coinfected HIV patients [6–8]. Considering this, the Centers for Disease Prevention and Control (CDC) recommends universal screening and vaccination of people with HIV against HBV and screening for HCV as of 2020 [9]. However, this protocol has generally not been well-practiced in many LMICs.

Even though Ethiopia is endemic to viral hepatitis, there is no national strategy for surveillance, prevention and control of the disease. Data that could guide practical interventions at a policy level are limited on these infections including among specific high-risk groups such as HIV-infected individuals. According to a systematic review of published studies, 5.2% and 5.5% of the HIV-infected individuals had HBV and HCV coinfection in Ethiopia, respectively [10]. However, updated evidence is required from different parts of the country as the risk factors and disease burden could vary geographically and with time. In addition, the effect of HBV and /or HCV coinfections on important treatment outcomes such as HIV-1 viral load suppression remains poorly defined, particularly in resource-limited areas. Therefore, this study was designed to determine the seroprevalence of hepatitis B and C viruses and assess their associations with HIV-1 viral load suppression among HIV-1 infected patients on ART at Mekelle hospital in northern Ethiopia.

## Methods

### Study design and setting

This cross-sectional study was conducted at the ART clinic of Mekelle General Hospital in northern Ethiopia, between February and April 2020. The hospital is located in Mekelle city, the capital of Tigray Regional State, which is located 783 Kilometers away from Addis Ababa. The hospital has a huge patient flow from the whole Tigray region, and neighboring regions of Amhara and Afar. Mekelle General Hospital offers a range of clinical services for its clients including voluntary counseling and testing for HIV, ART, and Prevention of Mother to Child Transmission. During our study, 4,480 patients were receiving ART service at the hospital. Most of the laboratory investigations on this study were performed at Tigray Health Research Institution (THRI) located in the same city.

### Sample size determination

The sample size for this study was calculated using a single proportion formula, n1 = z2p (1-p)/d2, where n1 was the initial sample size, with a confidence level of 95%, an estimated HIV-HBV coinfection rate of 11.7% [11], and a precision of 3%. After considering a 10% non-response rate, and applying a finite population correction, n2 = n1/(1 + (n1/N)), where N was the total number of HIV patients under ART at the study site, we obtained a final sample size of 439.

### Study populations and recruitment

All HIV patients on ART were our study populations. Providing written informed consent or assent, being an age of ≥ 3 years, and being on ART for at least 6 months at the start of the study were the inclusion criteria. Patients who were critically ill, those vaccinated for HBV, and those treated for HBV and/ or HCV were excluded. Finally, 439 patients were enrolled in the study conveniently. Subsequently, data on socio-demographic and clinical characteristics of the study participants were collected using a structured questionnaire and a standardized recording format, respectively.

### Sample collection and laboratory analysis

From each participant, 5 ml of venous blood was drawn aseptically. A rapid screening was done for HBV surface antigen (HBsAg) and anti-HCV antibodies using immunochromatographic assay on the plasma sample as per the manufacturer's instruction. The remaining samples were packed in a leak-proof triple container and transported to THRI at room temperature on the same day of collection.

Samples, which were positive for HBsAg and anti-HCV on the immunochromatographic assay, were confirmed with HBsAg and anti-HCV Enzyme-Linked Immuno-sorbent assay (ELISA) (Beijing Wantai Co. China) at THRI, respectively. The HBsAg/anti-HCV ELISA is a highly specific and sensitive fourth-generation qualitative serological test that detects both antigens and antibodies. ELISA tests were performed under sterile conditions. Positive and negative controls were used and the cut-off values for the respective tests were defined according to the manufacturer’s instructions.

HIV-1 RNA viral load was quantified using reverse transcription PCR. Briefly, HIV-1 RNA was extracted from 0.2 ml of plasma sample on the Abbott m2000sp automated sample preparation system (Abbott Molecular, USA). Extracted RNA was then measured using Abbott m2000rt quantitative Real-Time HIV-1 assay (Abbott Molecular, USA) with HIV-1 RNA detection level of 40 to 10 million copies/ml based on the manufacturer's procedures.

### Data analysis

Data was entered and analyzed using the SPSS version 25 statistical software. Our primary outcome was quantifying the seroprevalence of HBV and/or HCV among HIV-1 patients on ART at the study site. Comparing the HIV-1 viral load suppression rate between the HIV-HBV, and HIV-HCV coinfected and their respective non-infected groups was our secondary outcome variable. HIV-1 viral load level was categorized as suppressed if patients had a viral load of < 1000cps/ml and not suppressed for those with a viral load level of ≥ 1000 cps/ml at the 6 month of ART treatment as per the WHO guidelines.

Bivariate and multivariate logistic regression analyses were performed to examine the association of potential predictors with the respective outcome variables. Covariates with a p-value of ≤ 0.25 in the bivariate analysis and a collinearity matrix index of ≤ 0.7 were considered for inclusion in the multivariate model in both cases. Briefly, age categories, gender, marital status, educational level, baseline CD4 count, history of tattoo use, and having a history of sexually transmitted infections and multiple sexual partners were screened for inclusion in the HBV infection statistical model. For the HCV infection, only gender, occupation, a history of having chronic non-communicable diseases, and multiple sexual partners were screened. Age categories and some other potential predictor variables were not added to the HCV model because of the small positivity rate with only 16 participants being positive for HCV. In the HIV-HBV and HIV-HCV vs HIV-1 viral load suppression model, we added HBV status, HCV status, gender, baseline CD4 count in cells/ul, baseline HIV-1 viral load, and current ART adherence level.Comparisons between subgroups were expressed as odds ratios (OR) with a 95% confidence interval (CI). A p-value of ≤ 0.05 was used to declare a statistical significance. Patients' ART adherence level was duly considered when assessing the relation of HBV, and HCV coinfections with HIV-1 viral load suppression rate. Adherence level was calculated by dividing the number of doses of ART taken to the number of prescribed doses of ART and was expressed in percentage. It was then categorized as good adherence (> 95%), fair adherence (85–95%), and poor adherence (< 85%) as per the WHO standard guidelines.

## Results

### Characteristics of the study population

The baseline characteristics of the participants are shown in Table 1. Briefly, of the 439 HIV patients enrolled, 284 (64.7%) were females and 155 (35.3%) males with a mean age of 43 ± 13 years. More than half of the participants 233 (53.1%) were in the age group of 36–52 years whereas 30 (6.8%) of them were between 3 and 18 years old. The majority of the participants were urban dwellers 403 (91.8%) and Orthodox Christians 414 (94.3%). One hundred twenty-two (27.8%) of the participants did not have formal education. About one-third of the participants, 147 (33.5%) were on ART for 13 or more years. The majority of the participants 403 (91.8%) were on the first-line antiretroviral drugs and 326 (74.3%) had good adherence to the ART. The HIV-1 viral load level was found to be suppressed in 396 (90.2%) of the participants.

**Table 1:** Baseline characteristics of participants enrolled in a study for determining the seroprevalence of HBV and HCV coinfections among HIV-1 infected patients on ART at Mekelle hospital in northern Ethiopia (n = 439)

Characteristic	n (%)
Gender	
Male	155 (53.3)
Female	284 (64.7)
Age group in years	
3–18	30 (6.8)
19–35	89 (20.3)
36–52	233 (53.1)
≥ 53	87 (19.8)
Length of time on ART in years	
0.5–3	43 (9.8)
4–6	62 (14.1)
7–9	75 (17.1)
10–12	112 (25.5)
≥ 13	147 (33.5)
Current ART regimen^a^	
1st line regimen	403 (91.8)
2nd line regimen	36 (8.2)
Educational status	
Primary education	173 (39.4)
Secondary education	86 (19.6)
Illiterate	126 (28.7)
Tertiary education	51 (11.6)
Not applicable^b^	3 (0.7)
Occupation	
Employed	99
Merchant	81
Farmer	25
House wife	100
Others^c^	134
Residence	
Urban	403 (91.8)
Rural	36 (8.2)
Marital status	
Married	162 (36.9)
Single	74 (16.8)
Widowed	86 (19.6)
Divorced	85 (19.4)
Not applicable^d^	32 (7.3)
Current ART adherence level	
Poor	34 (7.7)
Fair	79 (18.0)
Good	326 (74.3)
Current HIV-1 viral load suppression level	
< 1000 cps/ml^e^	396 (90.2)
≥ 1000 cps/ml	43 (9.8)

*ART* antiretroviral therapy, *HIV* human immunodeficiency virus; *a *drug combinations & dosage depends on patients' factors, *b* for the 3 years old participants, *c* miscellaneous such as daily laborers, students and those without job, *d* includes participants with < 18 years old, *e* participants with suppressed viral load level

### Seroprevalence of HBV and HCV

Overall, 60 of the 439 (13.6%) participants were coinfected with either HBV or HCV. Specifically, the seroprevalences of HBV and HCV were 10% (44/439) and 3.6% (16/439), respectively. None of the participants were coinfected with both HBV and HCV.

### Relation of potential factors with HBV and HCV coinfections

The relationship of the potential factors with HBV infection is shown in Table 2. In the multivariate analysis, being illiterate (AOR = 6.57; 95% CI 1.04–41.6), and having a history of sexually transmitted infections (AOR = 4.44; 95% CI 1.31–15.0) and multiple sexual partners (AOR = 29.9; 95% CI 7.82–114.8) were strongly associated with HBV infection. In addition, participants with a baseline CD4 count of < 200 cells/ul (AOR = 18.79; 95% CI 2.18–161) were more likely to be infected with HBV. There was no significant difference by gender of the participants.

**Table 2:** Factors related to HBV infection among HIV-infected patients on ART at Mekelle hospital in northern Ethiopia in the bivariate and multivariate logistic regression analysis (n = 439)

Characteristic	HIV-HBV coinfection		COR (95% CI)	P value	AOR (95% CI)	P value
	Yes (n = 44)	No (n = 395)				
	n (%)	n (%)				
Gender						
Male	17 (10.9)	138 (89.1)	1.17 (0.62–2.23)	0.62	–	
Female	27 (9.5)	257 (90.5)	Ref			
Age group in years						
3–18	2 (6.7)	28 (96.7)	Ref			
19–35	7 (7.9)	82 (92.1)	1.19 (0.23 -6.09)	0.82	–	
36–52	25 (10.7)	208 (89.3)	1.68 (0.38–7.49)	0.68	–	
≥ 53	9 (10.3)	78 (89.7)	1.61 (0.33–7.93)	0.55	–	
Educational level*						
Primary education	16 (9.3)	157 (90.7)	2.65 (0.59–11.9)	0.21	3.15 (0.51–19.4)	0.22
Secondary education	3 (3.5)	83 (96.5)	0.94 (0.15–5.81)	0.97	0.75 (0.072–7.84)	0.81
Illiterate	23 (18.3)	103 (81.7)	5.81 (1.32–25.5)	0.02	6.57 (1.04–41.6)	0.04
Tertiary education	2 (3.9)	49 (96.1)	Ref		Ref	
Marital status^#^						
Married	13 (8.1)	149 (91.9)	0.84 (0.32–2.19)	0.72	–	
Divorced	12 (16)	73 (84)	1.75 (0.64–4.53)	0.28	–	
Widowed	11 (12.8)	75 (87.2)	1.41 (0.51–3.83)	0.51	–	
Single	7 (9.4)	67 (90.6)	Ref			
ART adherence level						
Poor	5 (14.7)	29 (85.3)	1.58 (0.57–4.37)	0.37	–	
Fair	7 (8.9)	72 (91.1)	0.89 (0.37–2.11)	0.79	–	
Good	32 (9.8)	294 (90.2)	Ref			
History of STIs						
Yes	16 (58.6)	41 (41.4)	4.93 (2.46–9.87)	< 0.001	4.44 (1.31–15.0)	0.041
No	28 (7.3)	354 (92.7)	Ref		Ref	
Tattoo use						
Yes	11 (11.8)	69 (86.2)	1.58 (0.35–7.10)	0.26	–	
No	33 (9.2)	326 (90.8)	Ref			
Baseline CD_4_ count (cells/ul)						
< 200	34 (30.3)	78 (69.7)	7.19 (1.63–31.6)	0.009	18.79 (2.18–161)	0.008
201–500	6 (2.5)	230 (97.5)	0.43 (0.083–2.22)	0.31	0.78 (0.086–7.18)	0.83
≥ 501	2 (3.5)	54 (96.5)	0.61 (0.082–4.54)	0.63	2.13 (0.15–29.7)	0.57
Test & treat	2 (5.7)	33 (94.3)	Ref		Ref	
History of multiple sexual partners						
Yes	16 (51.6)	15 (48.4)	16.3 (6.80–38.9)	0.001	29.9 (7.82–114.8)	< 0.0001
No	28 (6.8)	380 (93.2)	Ref		Re	

*AOR* adjusted odds ratio, *ART* anti-retroviral therapy, *COR* crude odds ratio, *CI* confidence interval, *HBV* hepatitis B virus, *HIV* human immune deficiency virus, *Ref* reference, *STIs* sexually transmitted infections, WHO world health organization;

^*^participants with non-applicable educational status were excluded from this analysis (n = 3); # = participants with non-applicable marital status were excluded from this analysis (n = 32)

Similarly, a multivariate analysis was performed to explore the predictor variables associated with HCV infection (Table 3). Briefly, participants with a history of sexually transmitted infections (AOR = 5.21, 95% CI: 1.39–19.5), and those reporting a history of chronic non-communicable diseases (AOR = 10.6, 95% CI 1.61–70.1) were more likely to be infected with HCV.

**Table 3:** Factors related to HCV infection among HIV-infected patients on ART at Mekelle hospital in northern Ethiopia in the bivariate and multivariate logistic regression analysis (n = 439)

Characteristic	HIV-HCV co-infection		COR (95% CI)	P value	AOR (95% CI)	P value
	Yes (n = 16)	No ( n = 423)				
	n (%)	n (%)				
Gender						
Male	7 (4.5)	148 (95.5)	1.45 (.53–3.96)	0.47	–	
Female	9 (3.2)	275 (96.8)	Ref			
Occupation						
Employed	2 (1.0)	97 (99.0)	0.25 (0.02–4.1)	0.32	–	
Merchant	3 (3.7)	78 (96.3)	0.61 (0.05–6.99)	0.68	–	
Others^a^	6 (4.5)	128 (95.5)	0.93 (0.11–8.31)	0.94	–	
House wife	4 (4.0)	96 (96.0)	1.81 (0.21–15.3)	0.58	–	
Farmer	1 (4.0)	24 (96.0)	Ref			
History of CNCDs						
Yes	2 (12.5)	14 (97.5)	3.88 (0.81–18.6)	0.09	10.6 (1.61–70.3)	0.014
No	14 (3.3)	408 (96.7)	Ref		Ref	
History of STIs						
Yes	6 (10.5)	51 (89.5)	4.37 (1.53–12.5)	0.006	5.21 (1.39–19.5)	0.014
No	10 (2.6)	372 (97.4)	Ref		Ref	

*AOR* adjusted odds ratio, *CNCDs* chronic non-communicable diseases, *COR* crude odds ratio, *CI* confidence interval, *HCV* hepatitis C virus, *HIV* human immune deficiency virus, *Ref* reference, *STIs* sexually transmitted infections, *WHO* world health organization, *a* miscellaneous such as daily laborers, students and those without job

### Hepatitis B and C viral coinfections and HIV-1 viral load suppression

Table 4 shows the effect of HBV and HCV coinfections on HIV-1 viral load suppression rate after adjusting for baseline viral load, adherence level, baseline CD4 T cell count, and other potential confounders. Briefly, there was no significant association between HBV infection status and HIV-1 viral load suppression rate. However, HCV infection status was significantly associated with variations in the viral load suppression rate where participants infected with this virus were more likely to have a higher viral load compared to the non-infected groups (AOR = 7.14; 95% CI 2.18–23.3). Participants with a higher baseline HIV-1 viral load were significantly more likely to have a higher viral load. Nonetheless, there was no association between ART adherence level and HIV viral load suppression. There was no significant difference in the viral load suppression rate between males and females.

**Table 4:** HIV-HBV, and HIV-HCV coinfections and their association with HIV viral load suppression among HIV-1 infected patients on ART at Mekelle Hospital in northern Ethiopia in the bivariate and multivariate logistic regression analysis (n = 439)

Variables	Current HIV-1 Viral Load Level		COR (95% CI)	P value	AOR (95% CI)	P value
	< 1000 cps/ml* ( n = 396)	> 1000 cps/ml (n = 43)				
HBV status						
Negative	356 (90.1)	39 (9.9)	Ref		–	–
Positive	40 (90.9)	4 (9.1)	0.91 (0.31–2.68)	0.86	–	–
HCV status						
Negative	387 (91.5)	36 (8.5)	Ref		Ref	
Positive	9 (56.2)	7 (43.8)	8.36 (2.9–23.7)	< 0.0001	7.14 (2.18–23.3)	0.001
Gender						
Female	262 (92.3)	22 (7.7)	Ref			
Male	134 (86.5)	21 (13.5)	1.86 (0.99–3.51)	0.053	2.11 (1.07–4.18)	0.31
Baseline CD4 count in cells/ul						
< 200	40 (10.1)	11 (25.6)	0.75 (.25–2.27)	0.61	0.77 (0.03–18.68)	0.87
201–500	197 (49.7)	14 ( 32.6)	2.91 (1.03–8.17)	0.04	3.32 (0.13–83.7)	0.46
≥ 501	130 (32.8)	12(27.9)	2.24 (0.77–6.46)	0.14	2.53 (0.09–64.4)	0. 57
Test and treat	29 (7.3)	6 (14.0)	Ref		Ref	
Baseline HIV-1 viral load						
< 1000cps/ml	368 (97.6)	9 (2.4)	Ref		Ref	
≥ 1000cps/ml	28 (45.1)	34 (54.9)	49.65 (21.67–113.7)	< 0.0001	52.6 (23.84–116.5)	0.001
Current ART adherence level						
Poor	28 (82.4)	6 (17.6)	Ref		Ref	
Fair	71(89.9)	8 (10.1)	0.53 (0.16–1.65)	0.27	0.63 (0.19–1.87)	0.29
Good	297 (91.1)	29 (8.9)	0.46 (0.17–1.19)	0.11	0.57 (0.21–2.23)	0.16

*AOR* adjusted odds ratio, *COR* crude odds ratio, *CI* confidence interval, *HBV* hepatitis B virus, *HCV* hepatitis C virus, *HIV* human immune deficiency virus, *Ref* reference, *WHO* world health organization

^*^a viral load of < 1000 cps/ml was the referent

## Discussion

In this study, we found that 10% and 3.6% of the HIV patients on ART were coinfected with HBV and HCV, respectively. Being illiterate, history of having multiple sexual partners, and reduced baseline CD4 T cell count were statistically associated with HBV infection. On the other hand, having chronic non-communicable diseases was statistically related to HCV infection. Participants with a history of sexually transmitted infections were more likely to be infected with either HBV or HCV. On further analysis, HCV infection status was significantly associated with decreased viral load suppression rate whereas no significant association was observed with the HBV infection. There were no significant differences in HBV and HCV seroprevalence and/ or in the viral load suppression rate between males and females.

The observed HBV infection rate (10%) is high and corresponds to a peak HBsAg prevalence of 10.8% observed among communities in Ethiopia earlier in 1986 [12]. Our figure is higher than the findings of similar studies in Southern Ethiopia (6.3%) [13], Gondar (5.6%) [11], a former study from the same study site (5.9%) [14], a national urban-based survey among adults (4.8%) [15], and a meta-analysis pooled estimate of 5.2% [10] in Ethiopia. While the sample size and type of diagnostic tools used in the previous studies are comparable with ours, study time and participants' behavioral differences could be attributed to the discrepancies. With the improved survival of HIV patients due to ART [16], there could be a continued transmission of HBV among our study participants over time, particularly given the absence of routine HBV screening and vaccination, and the risky behaviors of the participants. In our study, 59% of the participants were on ART for ten or more years and 36.4% of the HBV cases were individuals with multiple sexual partners. On the other hand, our finding is comparable with the results of a recent study from Eastern Ethiopia, which reported an HBsAg prevalence of 11.7% [17]. Comparable figures were also reported elsewhere in Kenya (10.3%) [18], Burkina Faso (10.4%) [19], and Zambia (11.3%) [20]. In contrast, the coinfection rate obtained in the present study is lower than a pooled estimate of 15% HBsAg prevalence in sub-Saharan Africa [5]. One possible explanation for this variation could be the difference in HIV and HBV burden across the region where countries like South Africa have a higher incidence of HIV than Ethiopia and thus potentially inflating the sub-Saharan HBV coinfection estimate [21].

The 3.6% HCV coinfection rate in our study is broadly comparable with the findings of several studies in Ethiopia, which reported coinfection rates ranging from 3.0 to 3.6% [13, 22]. However, higher rates of HCV coinfection were reported in a meta-analysis (5.5%) [10], a study from Gondar (5.0%) [11], and an earlier study from the same study site (9.2%) [23]. Differences in sample size and types of diagnostic tests used could be possible reasons for the variations. Briefly, the earlier study from the same study site used a 50% smaller sample than ours while the study from Gondar did not confirm the rapid test results with the more specific ELISA assay [10, 23]. Whereas our figure is lower than a pooled estimate of HCV infection among the general population in sub-Saharan Africa (7%) [5], it is only slightly higher than the pooled estimate for Ethiopia (3.1%) [10]. Much higher coinfection rates (30–50%) have been reported from some industrialized countries, such as North America and Europe, where intravenous drug use (IVDU) is a major risk factor for both infections [24]. Even though the main mode of HCV transmission in Africa has not yet been well established, IVDU appears to be less important than in Western countries [25]. In our study, it was difficult to identify a single influential mode of transmission for HCV though the majority of the patients reported HIV acquisition via sexual contact suggesting a possible co-transmission of both viruses.

In a multivariate analysis, illiterate participants were more likely to be infected with HBV as compared to the other groups, which is consistent with previous findings [26, 27]. This could indicate gaps in the knowledge and practice of HBV prevention in this specific group. These populations are also often with low standards of socioeconomic status and tend to have poor healthcare access. Having a history of sexually transmitted infections was also strongly associated with HBV or HCV infection. The fact that HIV and these two hepatitis viruses share the same routes of transmission supports this elucidation [5]. In African countries, HBV acquisition is assumed to occur mainly during early childhood, but heterosexual exposure is also cited as an important route of transmission [25]. In addition, participants with a baseline CD4 count of < 200 cells/ul) had significantly higher odds of having HBV infection than the other groups. Spontaneous clearance of HBV is low in individuals with reduced CD4 levels leading to persistent HBV infections [28]. However, the relationship could be reciprocal as HBV infection may lead to reduced CD4 + T cell level [7, 29]. Participants with a history of chronic non-communicable diseases were also more likely to be infected with HCV.

Furthermore, our findings demonstrated that participants with HIV-HCV infection had a decreased viral load suppression rate than monoinfected patients. Our results are consistent with findings of a cohort study from Spain, which showed a poorer virological response at 48 weeks from ART initiation in HCV-HIV coinfected compared to monoinfected patients [6]. However, it is worthy to mention that several studies have not found statistically significant differences in virological responses to ART by HCV infection status [30–32]. Many possible reasons including social, behavioral, and biological factors have been proposed for the poor virological responses to ART in HCV-HIV-coinfected individuals. In the current study, we adjusted the effect to the baseline HIV viral load level, ART adherence status, HBV coinfection, and CD4 T cell count level. The relationship between non-adherence to ART and poor HIV viral load suppression is a well-documented incident [33]. In this study, there was no association between ART adherence level and HIV viral load suppression. This could be because the majority (92.3%) of our study participants had a good to fair adherence to ART. The viral load suppression level in this study was also quite high with 90.2% of the participants achieving the suppression. However, Ethiopia lags in achieving the 90–90-90 end HIV/AIDS epidemic target where only an 84% of people living with HIV knew their status, 78% of people with HIV were on treatment and only 75% achieved viral suppression by 2021 [34]. Adherence is important to attain viral suppression and all ART regimens require at least 85% adherence level even though it does not necessarily prevent the accumulation of antiviral drug resistance mutations [35, 36].

The biological mechanism of the effect of HCV coinfection on ART response is complex, but a study suggests that HCV core could significantly enhance HIV replication in human macrophages by upregulating Tumoral Necrosis Factor and interleukin-6 [37]. On the other hand, we found no association between HBV-HIV coinfection and virological suppression, which is consistent with several studies [38, 39]. On the contrary, a study involving a large cohort of African patients reported a significant association between HIV-HBV co-infection and viral load suppression [40]. In general, the differences in the impacts of HCV and HBV on ART response could partially be attributed to variations in study design, sample size, and study participants.

The findings of our study have important implications for clinical and public health policy. The high HBV and substantial HCV coinfection rates in the present study suggest the need to adapt the internationally recommended protocols against viral hepatitis and HIV in Ethiopia. Knowledge of the patients' HBV-HCV status can help clinicians interpret the medical profiles effectively and guide decisions on the choice of best antiretrovirals for coinfected patients. For example, lamivudine is one of the drugs used as part of the first-line ART regimen in Ethiopia and has been approved for the treatment of chronic HBV. However, treating HIV patients coinfected with HBV using lamivudine may induce resistance to HBV as indicated in some studies with 71% of the patients developing resistance after five years of use of lamivudine [41, 42]. Besides, there could be a 'flare-up’ of HBV when the drug is stopped [43]. People with HIV who test positive for HBV should, therefore, receive HIV antiviral medication with activity against HBV such as tenofovir and entecavir [44]. Treatments are available for HCV but the high costs limit their use in most African settings including Ethiopia.

Much attention should also be given to the prevention of these viral infections in Ethiopia. Patients who are HBV- or HCV-infected and those at risk should be informed about transmission routes and methods to prevent a further spread of the viruses. Moreover, unvaccinated patients who test HBV-negative should be given a vaccine to prevent future infection. However, such a strategy is not possible for HCV, as a vaccine does not exist so far.

Although this study has generated valuable evidence on HIV and hepatitis viral coinfections, it was not without limitations. First, as we used HBsAg as a surrogate marker for HBV infection, some patients with occult HBV infections (positive HBV DNA in the absence of a positive HBsAg) could have been missed. However, the prevalence and clinical relevance of occult HBV in HIV-positive patients remain controversial. In contrast, the actual prevalence of HCV might be overestimated as we used a serology without HCV RNA confirmation. Even though spontaneous clearance of HCV is believed to be low among HIV/HCV coinfected patients, some patients could have been wrongly classified as HIV/HCV coinfected [45].

## Conclusions

The HBV coinfection rate in our study is high and corresponds to a hyperendemic level when gauged against the WHO's criteria. The HCV coinfection rate is also substantially high and urges attention given its influence on the viral load suppression of HIV patients on ART at our study site. Our findings suggest the need to adopt universal screening and vaccination of people with HIV against HBV and screening for HCV at our study site and in Ethiopia at large, which could enhance Ethiopia's progress towards the 2030 global target of reducing HBV infection.

## Data Availability

The datasets generated and/or analyzed during the current study are not publicly available because of the sensitive nature of the data but are accessible from the corresponding author on a reasonable request.

## Abbreviations

ART: Antiretroviral therapy
AIDS: Acquired immunodeficiency syndrome
CD4: Cluster of differentiation 4
ELISA: Enzyme-Linked Immuno-sorbent assay
HBsAg: Hepatitis B surface antigen
HBV: Hepatitis B virus
HCV: Hepatitis C virus
HIV: Human Immunodeficiency Virus
RNA: Ribonucleic acid
RT-PCR: Reverse transcription polymerase chain reaction
WHO: World Health Organization
